# *Salmonella* Serovar Wiki: a curated web portal providing a comprehensive summary of *Salmonella* serovars for academia, industry, and public health

**DOI:** 10.1128/msphere.00455-25

**Published:** 2025-10-20

**Authors:** Linghuan Yang, Caroline R. Yates, Phutthaphorn Phaophu, Martin Wiedmann, Renato H. Orsi

**Affiliations:** 1 Department of Food Science, Cornell University 5922, Ithaca, New York, USA; 2 Center for Advanced Therapeutics, Institute of Molecular Biosciences, Mahidol University 98841, Nakhon Pathom, Thailand; US Food and Drug Administration, Silver Spring, Maryland, USA

**Keywords:** *Salmonella*, serovar, public health, epidemiology, food microbiology, foodborne outbreaks

## Abstract

*Salmonella* is a major foodborne pathogen causing substantial illness and economic burden globally. To address the lack of a centralized resource, we developed the *Salmonella* Serovar Wiki, a curated web portal providing comprehensive, serovar-specific information on more than 100 *Salmonella* serovars, including their geographical distribution, genomic characteristics, animal reservoirs, and outbreak history. Built on the Confluence platform, this Wiki consolidates data from sources such as the European Center for Disease Prevention and Control, Centers for Disease Control and Prevention, the U.S. Food and Drug Administration, and Rapid Alert System for Food and Feed, offering a valuable tool for academia, industry, and public health professionals.

**IMPORTANCE:**

By facilitating rapid access to serovar-specific information, the *Salmonella* Serovar Wiki aids both epidemiologists and environmental health professionals in hypothesis generation, outbreak investigations, and research into understudied serovars, ultimately enhancing food safety and public health outcomes.

## INTRODUCTION

*Salmonella* is a major foodborne pathogen worldwide. The genus *Salmonella* consists of two species: *Salmonella bongori* and *Salmonella enterica* (1). Within *S. enterica*, there are six subspecies: subspecies *enterica* (I) (hereafter referred to as *S*.), subspecies *salamae* (II), subspecies *arizonae* (IIIa), subspecies *diarizonae* (IIIb), subspecies *houtenae* (IV), and subspecies *indica* (VI). However, at least one study has proposed a total of 11 *S*. *enterica* subspecies, including five novel designations (VII–XI), based on a large-scale analysis of whole-genome sequencing data (2). To date, more than 2,500 serovars have been identified across *Salmonella* (3). These serovars can be further classified into typhoidal/paratyphoidal *Salmonella* serovars, which cause enteric fever, and non-typhoidal *Salmonella* (NTS) serovars, which primarily cause intestinal inflammation (4). NTS serovars contribute to 11% of all foodborne illnesses and are the leading cause of death and hospitalization related to foodborne diseases in the United States (5). More specifically, in the United States, NTS infections account for an estimated 1.3 million illnesses, 12,500 hospitalizations, and 238 deaths every year (5). It has been estimated that invasive NTS disease accounts for 4.26 million disability-adjusted life-years (DALYs), while typhoidal salmonellosis is associated with 9.8 million DALYs globally (6). Typhoidal serovars such as *S*. Typhi and *S*. Paratyphi A cause an estimated 9.2 million cases of typhoid fever and 3.8 million cases of paratyphoid fever annually worldwide, resulting in approximately 133,000 deaths (7).

Several *Salmonella* serovars have been found to display distinct epidemiological and ecological characteristics. Some *Salmonella* serovars are host restricted or host adapted, while others can be found associated with a wide range of hosts (8). For instance, *S*. Typhi infects only humans, and *S*. Abortusovis is restricted to infections in sheep (9, 10). Ubiquitous serovars, such as *S*. Typhimurium, are classified as broad-host-range serotypes because they are frequently isolated from various species, including humans, livestock, domestic poultry, birds, and rodents (11). Host-adapted serovars, such as *S*. Dublin (adapted to cattle), *S*. Derby (adapted to swine), and *S*. Enteritidis (adapted to chicken), are generally associated with a specific host but can cause disease in other hosts (12). Therefore, *Salmonella* outbreaks linked to host-adapted serovars are more likely to be associated with foods related to the host to which the *Salmonella* is adapted. Jackson et al. concluded that over 80% of outbreaks associated with *S*. Enteritidis, *S*. Heidelberg, and *S*. Hadar were linked to poultry or eggs, while more than 50% of outbreaks involving *S*. Javiana, *S*. Litchfield, *S*. Mbandaka, *S*. Muenchen, *S*. Senftenberg, and *S*. Poona were associated with fresh produce (13). These authors also observed that *S*. Typhimurium and *S*. Newport were linked to various food vehicles (13). Certain *Salmonella* serovars show a restricted geographical distribution, while others are distributed globally. For instance, *S*. Agona is only prevalent in Latin America, North America, and Europe, while *S*. Rissen is common in Southeast Asia (14). Conversely, *S*. Enteritidis and *S*. Typhimurium are common on all continents.

Currently, no platform that collectively describes key *Salmonella*-related information at the serovar level, such as geographical distribution and genomic characteristics, has been implemented. Although a Quantitative Microbial Risk Assessment Wiki (https://qmrawiki.org/) has been implemented, it was not developed to fill this gap as (i) it includes less than 10 *Salmonella* serovars; and (ii) it primarily focuses on risk characterization (e.g., transmission routes, exposure assessment, and case fatality ratios). Thus, we developed the *Salmonella* Serovar Wiki, a platform that addresses the need for a centralized resource on *Salmonella* serovars that encompasses both well-studied and understudied serovars, with a commitment to maintaining actively updated information. The *Salmonella* Serovar Wiki can be used to support public health professionals, such as epidemiologists and environmental health experts, by facilitating hypothesis generation and aiding in outbreak investigations. Moreover, this Wiki can serve as a tool for industry to understand the contamination risks posed by specific *Salmonella* serovars. It provides key and relevant information on serovars of interest (e.g., historical outbreaks with case numbers and associated vehicles and genetic characteristics) that allows users to infer a serovar’s likelihood of causing human illness. The Wiki can also aid in internal traceback investigation and root cause analysis by identifying specific ingredients that have historically been contaminated with specific *Salmonella* serovars. Meanwhile, this Wiki enables academic researchers to quickly retrieve relevant information for a given *Salmonella* serovar, avoiding redundant searches.

## MATERIALS AND METHODS

### Selection of *Salmonella* serovars

Selection of *Salmonella* serovars for inclusion in the *Salmonella* Serovar Wiki followed a prioritization approach. The initial inclusion criteria focused on the 100 most common serovars in the NCBI Pathogen Detection (PD) database, based on the number of isolates for each serotype in the field “computed type.” The top 100 serovars were linked to 88% (*n* = 4,076) of the 4,619 *Salmonella*-associated outbreaks recorded in the National Outbreak Reporting System (accessed on 10 June 2025). Additional serovars were included if these serovars represented (i) recent outbreak-associated serovars and (ii) rare serovars flagged in recent border rejections via the Rapid Alert System for Food and Feed.

### Design of the *Salmonella* Serovar Wiki

The *Salmonella* Serovar Wiki offers key details about various *Salmonella* serovars, including (i) common sources typically associated with each serovar, (ii) the serovar geographical distribution, (iii) past outbreaks/recalls/border rejections associated with the serovar, (iv) association of serovar of interest with human illnesses, and (v) genomic characteristics of the serovar.

The web-based *Salmonella* Serovar Wiki is built on the Confluence platform. Accessing this Wiki does not require a login; however, making edits or contributions requires users to log in. Cornell-associated users can log in using their Cornell NetID account, while non-Cornell users must log in with a Confluence account. After logging in, all users must request the appropriate permissions from the *Salmonella* Serovar Wiki administrator to make edits. For further information, or if researchers wish to contribute to and update this Wiki, they should contact Martin Wiedmann at martin.wiedmann@cornell.edu.

### Structure and organization of the *Salmonella* Serovar Wiki 

The *Salmonella* Serovar Wiki comprises (i) a content page and (ii) individual pages with specific information for each serovar. The content page includes a brief introduction to the *Salmonella* Serovar Wiki as well as a list of serovars, in alphabetical order, included in this Wiki. Each serovar name is hyperlinked and redirects to the corresponding page for that serovar. For a given serovar, there are a total of eight sections presented in the following order: (i) Background Information, (ii) Genetic Characteristics, (iii) Animal Reservoir, (iv) Geographical Distribution, (v) Human/animal Outbreaks, (vi) Border Rejections, (vii) Recalls, and (viii) Relevant Links. A description of each section can be found in Table 1. At the end of the content page, a website map widget, ClustrMaps (https://clustrmaps.com/), is implemented to display the total page views and the locations of recent visitors (city and country, if available), and the number of visits per location. For privacy reasons, IP addresses are neither displayed nor collected by this Wiki. To account for the total number of page views, we excluded views from Ithaca, NY, as we are frequently updating the page from this location.

**TABLE 1:** Sections for a given serovar in the *Salmonella* Serovar Wiki

Section	Description
Background information	This section provides a brief description of the serovar, including the full scientific name. The antigenic formula and serogroup information are extracted from the scheme: Antigenic Formulae of the *Salmonella* Serovars (9th ed.) Paris: WHO Collaborating Centre for Reference and Research on *Salmonella*. When available, the following information is also included for each serovar: (i) the initial identification (time and location of first isolation), (ii) case reports describing associated clinical symptoms, and (iii) prevalence levels (non-human or clinical). A link to NCBI Pathogen Detection (PD) listing all isolates for the given serovar is included at the end.
Genetic characteristics	This section illustrates the antimicrobial resistance patterns for isolates within a given serovar when available, specifying whether they are multidrug resistant or pan-susceptible. It also includes studies that indicate whether isolates are hypervirulent, hypovirulent, or possess unique or characterized virulence factors. Phylogenetic information was incorporated from two studies conducted by our group (12, 15).
Animal reservoir	Any known animal reservoirs for this serovar, such as chicken and cattle, are listed. If unknown, host information is extracted from the “host” column in NCBI PD metadata and summarized into a broader animal category (e.g., *Bos taurus* and “bovine” would be listed as “cattle” in the Wiki).
Geographical distribution	This section describes the countries or regions where isolates within this serovar have been reported. Areas where a given serovar is commonly isolated are also documented. Information on geographical distribution is primarily retrieved from the sources listed in Table 2. These sources include the origin of foods in border rejections, the location of outbreaks, the origin of recalled foods, and scientific publications. If the distribution of a specific serovar is not detailed in Table 2, such information is obtained at the country level from the “location” column in the NCBI PD metadata. The geographical distribution is described as “global” or “worldwide” for serovars found across multiple continents.
Human outbreaks	Each human outbreak consists of the following information: (i) year, (ii) location, (iii) associated source, and (iv) number of cases. Human outbreaks are listed from the most recent to the oldest. If an outbreak involves multiple serovars or if the number of cases is associated with multiple countries, superscripts are used to provide explanations. Notably, certain entries such as two *S*. Gallinarum biovars—Gallinarum and Pullorum—are primarily associated with animal outbreaks. In these cases, only animal outbreaks are reported. If there are too many outbreaks to include for a serovar of interest, we first clarify this in the section and then select a few examples associated with the most common commodity, along with outbreaks linked to uncommon sources. If no recent human outbreaks have been reported, it is simply stated that there have been no recent outbreaks linked to this serovar.
Border rejections	Each border rejection includes:(i) year, (ii) exporting country, (iii) country issuing a border rejection, (iv) associated source, and (v) product category. If a border rejection is associated with multiple serovars or multiple countries enforcing border rejections, superscripts are added for clarification. If no recent border rejections have been reported, it is simply stated that there have been no recent border rejections linked to this serovar.
Recalls	Each recall is described with the following details: (i) year, (ii) location, (iii) recalled product, and (iv) type. If a recall involves multiple serovars, superscripts are applied to clarify. If no recent recalls have been reported, it is simply stated that there have been no recent recalls linked to this serovar.
Relevant links	All references and sources cited above are listed using numbered hyperlinks. For links that had expired, we used the Wayback Machine (https://web.archive.org/) tool to access archived copies of defunct web pages. The new links archived by the Wayback Machine are provided in this section.

### Sources used to update the *Salmonella* Serovar Wiki

The *Salmonella* Serovar Wiki consolidates information from publicly available data sources, including peer-reviewed scientific literature, surveillance reports, genomic databases, and regulatory agency publications. Multiple authoritative sources, including but not limited to U.S. entities such as the Centers for Disease Control and Prevention (CDC), the U.S. Food and Drug Administration, the NCBI PD database, the European Center for Disease Prevention and Control, and the World Health Organization (WHO), were extensively utilized to gather serovar-specific data. A specific list of resources along with descriptions can be found in Table 2.

**TABLE 2:** List of sources used to update the *Salmonella* Serovar Wiki^*b*^

Source	Division	Description
Food Safety News (https://www.foodsafetynews.com/)	NA^*c*^	Covers current foodborne illness outbreaks, recalls, and regulatory updates.
RASFF (16)	NA	When food safety issues arise, RASFF notifications are sent to alert relevant authorities. These alerts often lead to product recalls or border rejections, depending on the severity and geographical impact of the risk.
PubMed (17)	NA	On PubMed, relevant papers related to serovar of interest can be retrieved.
NCBI Pathogen Detection (PD) (https://www.ncbi.nlm.nih.gov/pathogens/)	NA	NCBI PD aggregates sequence data and metadata from sources worldwide, including but not limited to public health labs, government agencies, and research institutions, to identify potential clusters of infections and isolation sources, investigate links between cases, and assess AMR profiles.
Institut Pasteur, Antigenic Formulae of the *Salmonella* Serovars from the World Health Organization (18)	NA	This resource provides a detailed formula for each *Salmonella* serovar, e.g., listing its unique combination of antigens, including somatic (O), flagellar (H) antigens, capsular (Vi) antigens, as well as serogroup information.
CDC	*Salmonella* Atlas 1968–2011^*a*^ (19)	The Atlas provides information on animal reservoirs and geographical distribution of cases for a given serovar, but all results are based solely on U.S. isolates.
	Morbidity and Mortality Weekly Report (20)	These reports contain relevant information on outbreaks and isolation sources at the serovar level (U.S. only).
	Reports of Selected *Salmonella* Outbreak Investigations (21)	These reports summarize selected outbreak investigations linked to food, by year.
	Bacteria, Enterics, Ameba, and Mycotics Dashboard (22)	The dashboard provides AMR information for a given serovar, including the percentage of outbreak-associated isolates with clinically important AMR.
	FoodNet Fast (23)	Tracks infections caused by foodborne pathogens like *Salmonella*, *Campylobacter*, *Escherichia coli*, and others, in the U.S.
U.S. Department of Agriculture, FSIS	Quarterly Sampling Reports on *Salmonella* and *Campylobacter* (24)	These reports provide percentage of positive samples and serotype sampling data for *Salmonella* and *Campylobacter* for FSIS-inspected raw products.
	Outbreak Investigation Annual Reports (25)	These reports provide a summary of outbreaks investigated each fiscal year, detailing the number of outbreaks, pathogens involved, associated products, illnesses, and the number of outbreaks leading to product recalls.
	NARMS Multi-Year Report 2014–2019^*a*^ (26)	This report analyzed trends in *Salmonella* serotypes and AMR in select food animal species and products sampled through FSIS NARMS from 2014 to 2019.
FDA	Public Health Advisories from Investigations of Foodborne Illness Outbreaks (27)	These advisories are issued during outbreak investigations when specific, actionable steps are identified for consumers to protect themselves.
	NARMS Now (28)	This interactive FDA tool provides AMR data for bacteria isolated from humans, including *Salmonella*.
SPRAT (https://cse-sprat-dev-web-01.oit.umn.edu/)	NA	SPRAT is an interactive resource designed for monitoring the top food animal-related *Salmonella* serovars using publicly available sequence data from CDC and USDA-FSIS. It enables users to visualize the genomic landscape of each serovar, compare characteristics such as virulence and AMR, and identify new clusters.
ECDC	Surveillance Atlas of Infectious Diseases (29)	An interactive tool that provides access to the latest data on various infectious diseases, including salmonellosis, allowing users to explore and manipulate the information to generate custom tables and maps.
	Salmonellosis - Annual Epidemiological Report 2022^*a*^ (30)	This report focuses exclusively on salmonellosis and is based on 2022 data retrieved from The European Surveillance System, a platform for collecting, analyzing, and disseminating information on communicable diseases.
	The European Union Summary Report on Antimicrobial Resistance in Zoonotic and Indicator Bacteria from Humans, Animals and Food in 2021–2022^*a*^ (31)	This report, jointly produced by the European Food Safety Authority and ECDC, summarizes the key findings from the 2021–-2022 monitoring of AMR in *Salmonella* spp., *Campylobacter jejuni*, and *Campylobacter coli* in humans and food-producing animals, including laying hens, broilers, cattle under 1 year old, fattening turkeys, and pigs, as well as in the associated meat products.

^*a*^ All documents reflect the latest versions available at the time of publication. *Salmonella* Serovar Wiki updates its content to newer versions upon availability.

^*b*^ AMR, antimicrobial resistance; CDC, Centers for Disease Control and Prevention; ECDC, European Center for Disease Prevention and Control; FDA, Food and Drug Administration; FSIS, Food Safety and Inspection Service; NARMS, National Antimicrobial Resistance Monitoring System; PubMed, Public Medical Database; RASFF, Rapid Alert System for Food and Feed; SPRAT, *Salmonella* Predictive Risk Assessment Tool.

^*c*^ NA indicates not applicable.

### Browser compatibility testing

To guarantee cross-platform compatibility and support across various browsers, the *Salmonella* Serovar Wiki has been tested and is compatible with the three most popular desktop web browsers: Microsoft Edge (version 131.0.2903.86), Google Chrome (version 132.0.6834.83), and Mozilla Firefox (version 133.0.3). It has also been tested on two widely used mobile web browsers: Safari (version 18.1.1) and Samsung Internet (version 27.0.7.12).

### Future maintenance and curation of the *Salmonella* Serovar Wiki

Currently, the primary responsibility for the maintenance and curation of the *Salmonella* Serovar Wiki lies with members of the Cornell Food Safety Laboratory. However, we advertise the Wiki at conferences and invite collaborators (e.g., lab alumni who are already familiar with the Wiki and established researchers with research programs involving *Salmonella*) to recruit global volunteers to contribute to the Wiki. The goal is to update the *Salmonella* Serovar Wiki with additional serotypes as well as new information such as warnings from the Program for Monitoring Emerging Diseases, the Rapid Alert System for Food and Feed, and media outlets including Food Safety News. The goal is to add new outbreaks, recalls, or border rejections if they meet the following criteria: (i) they involve substantial public health concerns (e.g., a human outbreak with more than 100 cases); (ii) they are associated with new commodities not yet documented in our Wiki; or (iii) they provide final updates on previous, ongoing investigations.

## RESULTS

As of 1 July 2025, the *Salmonella* Serovar Wiki included detailed information on 110 *Salmonella* serovars, including the 100 most common *Salmonella* serovars available in the NCBI PD Database (15). The remaining 10 serovars were classified as non-top 100 and were relatively rare. Among the total of 110 serovars, 106 belong to *Salmonella enterica* subspecies *enterica* (I), one to subspecies *arizonae* (IIIa), one to subspecies *diarizonae* (IIIb), and two to subspecies *houtenae* (IV). Among the 110 *Salmonella* serovars, 107 are non-typhoidal and three are typhoidal. In total, 416 human outbreaks, 12 animal outbreaks, 159 border rejections, and 150 recalls are documented in the *Salmonella* Serovar Wiki for the 110 serovars included as of 1 July 2025.

**Fig 1:**
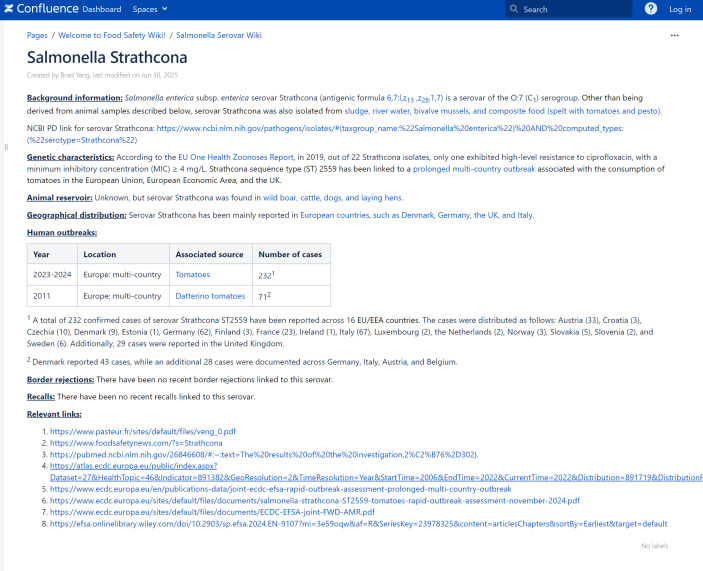
Example serovar entry: *Salmonella* Strathcona in *Salmonella* Serovar Wiki.

Each serovar entry includes detailed sections on its background, genetic characteristics, animal reservoir, geographical distribution, human or animal outbreaks, as well as information on border rejections and recalls, along with relevant external links where available. *S*. Strathcona, a rare serovar linked to a multicountry outbreak associated with tomatoes in Europe, is presented as an example entry in the *Salmonella* Serovar Wiki (Fig. 1).

Between 14 November 2024 and 1 July 2025, the *Salmonella* Serovar Wiki received a total of 1,376 views from 20 countries. The United States, Canada, and the Czech Republic were the top three countries, with the United States accounting for 770 views. These 770 views originated from 13 states, with New York, California, and South Dakota having the most views (395, 58, and 37 views, respectively).

## DISCUSSION

### Examples of how the *Salmonella* Serovar Wiki can help public health professionals during outbreak investigations

**Fig 2:**
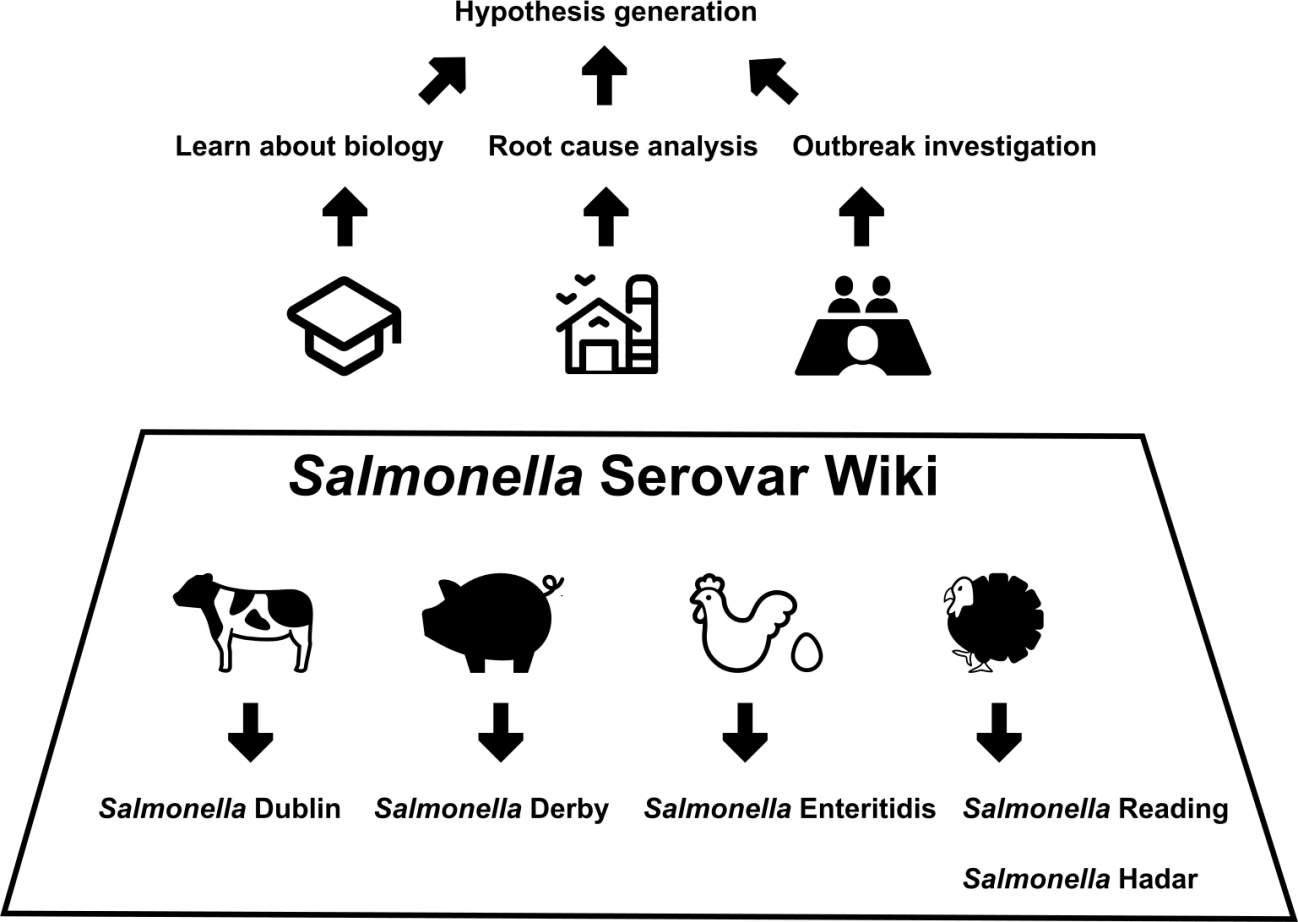
Schematic of how the *Salmonella* Serovar Wiki can support hypothesis generation in academia, industry, and public health using livestock-associated serovars as examples.

The *Salmonella* Serovar Wiki could be a resource for public health workers, aiding in the identification of outbreak sources and the development of effective strategies for prioritizing actions during outbreaks. More specifically, this Wiki can facilitate collaboration between epidemiologists and environmental health professionals (EHPs) while also supporting professionals working independently in either field. Epidemiologists can use the information available on this Wiki to (i) develop hypotheses on potential reservoirs and transmission pathways of specific serovars involved in new outbreaks and (ii) supplement standard surveys with additional targeted questions to identify the outbreak-associated vehicle (Fig. 2). For example, when *S*. Derby is isolated from patients in an ongoing outbreak, epidemiologists can find valuable information in the Animal Reservoir and Human Outbreaks sections of this Wiki, including (i) that *S*. Derby is host adapted to swine (32) and (ii) details of previous outbreaks associated with swine or swine products. In this scenario, epidemiologists carrying out interviews with cases could add and prioritize the question: “Have you consumed pork or pork-derived products in the past few days?”

On the other hand, EHPs can leverage information from this Wiki to (i) prioritize sampling targets (including potential animal reservoirs, environmental sources, and food products) typically linked to a given serovar and (ii) prioritize certain questions for worker interviews during outbreak investigations. For instance, when a dairy farm is linked to an outbreak associated with *S*. Dublin, EHPs can find in the Background section of this Wiki that salmonellosis caused by *S*. Dublin in cattle is usually associated with respiratory signs in calves (33, 34). Therefore, targeted sampling of the nostrils of dairy calves should be their top priority rather than sampling cattle feces. While the *Salmonella* Serovar Wiki is not intended to serve as a standard protocol for environmental sampling, it provides valuable supplemental information that, when combined with epidemiological evidence (e.g., interview data), facilitates EHPs to prioritize sampling sites with a higher probability of identifying the target serovar.

### Examples of how the *Salmonella* Serovar Wiki can help researchers in academia

For academia, the *Salmonella* Serovar Wiki provides scientists with serovar-specific information to (i) help generate hypotheses about specific serovars, (ii) understand the differences between serovars, and (iii) avoid undesirable search results. For example, researchers studying *S*. Amsterdam frequently encounter irrelevant results from *Salmonella* studies conducted in Amsterdam involving other serovars. Our platform solves this problem by aggregating curated key information for each serovar, enabling efficient access to relevant research while filtering out these confounding references. Furthermore, our Wiki highlights the importance of interpreting *Salmonella* serotype names, as the majority were named based on the location or symptoms in hosts where they were first isolated. As Gossner et al. suggested, *Salmonella* serotype names can be misleading, and researchers should be aware that a name does not necessarily indicate how common a serovar is in a particular region or whether it is linked to certain foods (35). The risk of getting *S*. Reading infection in Reading, England, is likely no greater than in Chicago, IL, USA. The Geographical Distribution section available on our Wiki may be valuable for researchers studying a new serovar, helping them avoid this confusion.

### Examples of how the *Salmonella* Serovar Wiki can help food safety professionals in the industry

The *Salmonella* Serovar Wiki supports industry root-cause analyses by linking contamination events to likely sources, e.g., implicating turkey as the probable origin when *S*. Reading or *S*. Hadar are detected in pasta meals containing turkey, given these serovars’ established association with turkey (12, 13). Retailers can then trace back through turkey suppliers to identify the potential contamination route. The platform further helps decision-making by providing access to regulatory actions (e.g., border rejections and recalls) and alternative re-purposing strategies documented for specific serovar-commodity combinations, such as Switzerland’s diversion of *S*. Livingstone-contaminated rapeseed meal to fuel production (16). If another food company finds *S*. Livingstone in their rapeseed products, they could use the information available on this Wiki to implement more cost-efficient measures, similar to those taken in Switzerland. The *Salmonella* Serovar Wiki includes hyperlinks to each resource, allowing users to explore further details as needed. This enables industry operators to evaluate appropriate mitigation measures—including chemical/physical treatments or product repurposing—based on the identified serovar, contamination level, and commodity characteristics, rather than relying solely on product detention or destruction following a positive *Salmonella* result.

The geographical distribution of serovars documented in our Wiki can reveal not only those that are distributed worldwide but also those reported exclusively in certain regions. For example, *S*. Napoli has only been found in the United States, Australia, and Europe, particularly in Italy (36–38). This information is valuable for identifying regions where the serovar of interest is frequently isolated. For instance, if a food company detects one *Salmonella* serovar in their final product (which contains mixed ingredients sourced from multiple countries), they could use this Wiki to trace the contamination source for that serovar. This includes identifying which ingredients are likely associated with a given *Salmonella* serovar and where that *Salmonella* serovar is most prevalent. Furthermore, this Wiki can help food safety professionals in the food industry to quickly assess the risk of ingredients from certain regions carrying specific serovars. If a particular food has been linked to multiple outbreaks in a country and a given serovar is consistently isolated from it, companies should evaluate whether importing that product poses potential contamination and health risks. By summarizing key information for each serovar, the *Salmonella* Serovar Wiki provides users with information to facilitate rapid hypothesis generation and the tracing of contamination sources for recalls, border rejections, and outbreaks.

### Bringing attention to rare *Salmonella* serovars: enhancing the *Salmonella* Serovar Wiki for broader research impact

Certain serovars such as *S*. Typhimurium and *S*. Enteritidis are heavily studied. For example, there are at least 43,188 papers available on PubMed (accessed on 25 August 2025) identified by searching for *S*. Typhimurium. *S*. Typhimurium is also known as a model serovar for studying *Salmonella* pathogenesis, with an extensive amount of research on its virulence and gene regulation available. However, the majority of *Salmonella* serovars are not well understood or studied by researchers. Their genetic traits, in particular, may still remain uncharacterized. For instance, a total of three papers were found to be associated with *S*. Vitkin as of 1 July 2025. By aggregating publicly available information on rare serovars into a single platform, we aim to make this Wiki more comprehensive, avoiding fragmented data scattered across the internet. A typical example from our Wiki is *S*. Strathcona, a rare serovar that has been infrequently associated with human outbreaks. One outbreak, in 2011, was a multicountry event in Europe, with Datterino tomatoes identified as the food vehicle, resulting in a total of 72 cases (39). A more recent *S*. Strathcona outbreak (2023–2024) was also linked to tomatoes, leading to 232 illnesses across Europe (40). These two outbreaks suggest that tomatoes may be a main food vehicle for *S*. Strathcona in Europe. If a future outbreak involving *S*. Strathcona occurs in Europe, investigators could use information from the Wiki to prioritize the investigation of tomatoes as a potential carrier based on these previous findings. Our hope is that by including these rare serovars, more researchers will focus on these understudied serovars.

### Limitations and future considerations of the *Salmonella* Serovar Wiki

Previous efforts have been made to facilitate hypothesis generation during outbreak investigations. In 2022, White et al. developed a prediction tool to trace potential transmission vehicles of *Salmonella* and Shiga toxin-producing *Escherichia coli* during outbreaks, utilizing outbreak data from the CDC’s Foodborne Disease Outbreak Surveillance System and Animal Contact Outbreak Surveillance System (41). While the Wiki and the outbreak source prediction tool both provide members of an outbreak investigation team with tools for hypothesis generation, these tools differ in their scope and intended use. The Wiki encompasses broader information for 110 serovars to provide a starting point for further investigation into each serovar and is not intended for usage as a prediction tool. One limitation of the Wiki is that the information for a serovar may not be specific enough for the Wiki to serve as the only tool for public health, research, or industry applications. Instead, the Wiki serves as a supplemental tool that consolidates a wide range of information to connect users to additional resources about each serovar and can be used in conjunction with other tools to assist public health, academia, and industry professionals. Users may need to use the relevant links for a given serovar to answer more specific questions, depending on their goal, such as analyzing serovar data from published studies or NCBI PD. The Wiki is also limited by the resources that are used for its updates. For example, when the resources listed in Table 2 provided insufficient information on geographical distribution or host, we used NCBI PD as a supplementary source. However, the data entry for the Host and Location sections of NCBI PD is non-standardized, which can pose challenges when summarizing these data. Additionally, the 10 sources that are used to update the Wiki are largely biased toward U.S.-based data. While the Wiki is also updated using sources with publicly available data from Europe and the WHO, it would further benefit from building a network of global contributors in the future. While current classification of *Salmonella* serovars remains based on serological properties, genome-based subtyping available within web-based platforms, such as Enterobase, may provide better resolution and representation of the natural populations of *Salmonella* (42). Nevertheless, it is unlikely that serotyping (or *in silico* serotyping) will stop being used to characterize *Salmonella* isolates, given the legacy knowledge associated with this subtyping method that has been used for decades.

The *Salmonella* Serovar Wiki aims to provide professionals in industry, academia, and government with up-to-date information on selected *Salmonella* serovars with high impact to food safety and public health. For researchers interested in learning or exploring *Salmonella* serovars, the *Salmonella* Serovar Wiki provides a reliable, concise overview of key information for each serovar by consolidating relevant data into a single, easily accessible platform, reducing the need for repeated searches through numerous papers and resources. Our team continuously updates the *Salmonella* Serovar Wiki by adding new serovars and more information. We encourage visitors to bookmark the Wiki and return frequently to access the newest updates.

## Data Availability

The Salmonella Serovar Wiki can be accessed at https://confluence.cornell.edu/display/FOODSAFETY/Salmonella+Serovar+Wiki.
